# Editorial: New potential biomarkers and cellular strategies for the study of prostate cancer and testicular cancer cells

**DOI:** 10.3389/fendo.2025.1737201

**Published:** 2025-11-13

**Authors:** Sabrina Bossio, Anna Grazia Recchia, Jorge Adrián Ramírez De Arellano Sánchez

**Affiliations:** 1 Department of Experimental and Clinical Medicine, University of Catanzaro “Magna Græcia, Catanzaro, Italy; 2 Independent Researcher, Toronto, ON, Canada; 3 Department of Molecular Biology and Genomics, Universidad de Guadalajara, Guadalajara, Mexico

**Keywords:** prostate cancer (PCa), testicular germ cell tumors (TGCTs), molecular mechanisms, precision medicine, tumor progression, epigenetic regulation, immunotherapeutic approaches

Prostate cancer (PCa) and testicular germ cell tumors (TGCTs) represent two distinct but clinically significant malignancies of the male genitourinary system. Despite differences in incidence, prognosis, and clinical course, both entities share common challenges in diagnosis, risk stratification, and therapeutic management. The identification of reliable biomarkers and the development of novel cellular strategies are urgently needed to improve early detection, personalize treatment approaches, and understand the underlying mechanisms responsible for disease resistance and progression. This Research Topic, *New potential biomarkers and cellular strategies for the study of prostate cancer and testicular cancer cells*, brings together nine articles that collectively illustrate the breadth of current research in this field, ranging from the identification of molecular and genetic biomarkers and systemic inflammation indices to the proposal of novel therapeutic approaches.

One current line of research highlighted in this Research Topic focuses on elucidating the molecular mechanisms of tumor development and progression. In their review, Xiang et al. discuss multifactorial pathways driving benign prostatic hyperplasia (BPH), emphasizing not only hormonal and inflammatory pathways but also the emerging roles of oxidative stress, autophagy, pyroptosis, and the mitochondrial antioxidant enzyme Prdx3. The authors draw attention to the paradox by which Prdx3, while reducing reactive oxygen species (ROS), inhibits autophagy and indirectly promotes oxidative damage, inflammasome activation, and pyroptosis, to ultimately maintain prostatic cell proliferation. This interplay reveals the limitations of conventional antioxidant therapies and suggests novel strategies targeting Prdx3, autophagy, and pyroptosis as potential avenues to control the progression of BPH.

Novel molecular markers were identified by Hou et al., who propose GPR137C as a potential novel biomarker linked to PCa progression. Using a combination of G-quadruplex sequence prediction and comprehensive bioinformatics analyses, the study demonstrates that the expression of GPR137C is strongly associated with tumor aggressiveness and poor prognosis. The authors propose that GPR137C could serve not only as a diagnostic and prognostic marker but also as a candidate therapeutic target, underscoring the utility of integrative computational approaches in the discovery of biomarkers.

The study by Wang et al. also emphasized the importance of epigenetic regulation in predicting outcomes. The researchers focused on the prognostic role of TET2 gene mutations in transition-zone PCa (TZ-PCa), a subtype often considered less aggressive than peripheral-zone PCa but currently unexplored at the molecular level. Using whole-exome sequencing of tumor samples from patients with TZ-PCa and the application of machine learning models, the team identified that TET2 mutations, a key enzyme involved in DNA demethylation and epigenetic regulation, were significantly correlated with worse clinical outcomes, including higher rates of biochemical recurrence and shorter progression-free survival, compared with tumors without such mutations. The findings suggest that loss of TET2 function may contribute to more aggressive tumor phenotypes in the transition zone, possibly through aberrant regulation of gene expression and tumor suppressor pathways. This study showed that TET2 mutations, detected by high-throughput sequencing, represent a valuable prognostic biomarker in TZ-PCa, highlighting the prognostic value of epigenetic alterations in PCa and suggesting that TET2 status could inform more precise risk stratification and patient management.

An important complementary contribution is the study by Sharma et al. In their review, the authors provide a comprehensive synthesis of how histone modifications, such as histone acetylation, methylation, phosphorylation, and ubiquitination, play critical roles in the pathogenesis and progression of PCa by modulating the chromatin state, transcriptional regulation, and key tumor-suppressor or oncogene networks. They emphasize that these histone modifications are emerging as diagnostic/prognostic biomarkers and may act as therapeutic targets (via HAT, HDAC, HMT, HDM inhibitors) in PCa. In particular, the review reinforces the notion that epigenetic dysregulation extends beyond DNA‐methylation to chromatin-level modifications, and that targeting the histone modification machinery might open new avenues toward precision oncology for PCa. Furthermore, their findings enrich the epigenetic biomarker dimension, linking the earlier studies on TET2 mutations with downstream chromatin remodeling events and thus rounding out the molecular biomarker landscape.

The translation of biomarker discovery into clinical practice is well represented by the contribution of Bugoye et al. Here, the authors describe how recurrent alterations such as loss of PTEN and the presence of the TMPRSS2:ERG fusion define distinct molecular subgroups of PCa, each with implications for therapy selection and disease monitoring. The emphasis on subtype-specific management further highlights the ongoing shift toward tailored oncological care.

Che et al. review the current state of immunotherapeutic approaches, including immune checkpoint inhibitors and vaccine-based strategies. Although immunotherapy has transformed outcomes in other solid tumors, its integration into PCa treatment still remains challenging. The authors address both the progress achieved and the barriers that remain to be overcome, pointing to combinatorial strategies as promising avenues.

Beyond tumor-intrinsic factors related to pathogenesis and treatment strategies, systemic biomarkers also provide valuable, non-invasive tools for risk assessment. Chen et al. contribute a systematic review and meta-analysis on the prognostic value of the Systemic Immune-Inflammation Index (SII) for patients undergoing radical prostatectomy, showing that higher SII levels are significantly associated with poorer oncological outcomes. Complementing this work, He et al. demonstrate, using data from the National Health and Nutrition Examination Survey (NHANES) 2001-2010, that an elevated SII correlates with increased PCa risk in the older patient population in the U.S., supporting the role of systemic inflammation in cancer susceptibility. Similarly, another study by Chen et al. explores the Hemoglobin, Albumin, Lymphocyte, and Platelet (HALP) score as a predictor of prostate-specific antigen levels and all-cause mortality. This study suggests that indices that integrate hematological and nutritional parameters can offer more information on prostate health and overall survival, even in individuals not diagnosed with PCa.

Altogether, these contributions illustrate the complexity of PCa biology and the diversity of approaches to the discovery of biomarkers and their implications for treatment. From intracellular pathways of oxidative stress and genetic mutations to epigenetic and chromatin-level alterations, from systemic inflammation indices to immunotherapeutic stratification, the Research Topic underscores the necessity of a multi-level perspective for addressing patients with PCa. In particular, although this Research Topic is focused primarily on PCa, the methodological approaches, such as sequencing, machine learning, chromatin analyzes, immunotherapy development, and systemic biomarker evaluation, are equally relevant to the study of TGCTs, where the search for prognostic markers and innovative therapeutic strategies remains ongoing.

This Research Topic highlights significant strides in understanding the molecular, cellular, and systemic dimensions of male genitourinary cancers. The included studies highlight the pivotal role of biomarker discovery, not only for diagnosis or prognosis, but for guiding precision medicine. In the future, the integration of multi-omics data (genomic, epigenomic, transcriptomic, proteomic), machine learning approaches, and robust clinical validation will be essential to translate these insights into tangible patient benefits. We extend our gratitude to all of the contributors and hope that this Research Topic inspires further advancements in biomarker-driven exploration and therapeutic innovation for prostate and testicular cancers.

